# Peroxide of Hydrogen

**Published:** 1896-09

**Authors:** 


### PEROXIDE OF HYDROGEN.

Dr. Warren Brown, of Tacoma, Washington, in a paper on "Peroxide of Hydrogen," read before the Washington State Medical Society, and published in the *Medical Sentinel*, of Portland, Ore., Feb. 1896, after alluding to its method of manufacture, speaks of it therapeutically as follows:

Gonorrhœa may often be aborted by using a full strength hydrogen dioxide injection immediately on the very first appearance of discharge. The injection should be used four to six times in twenty-four hours and retained for five minutes.

Cystitis, where pus is voided with the urine, often yields rapidly to injections of a solution containing two ounces to the pint.

Otitis media is treated by hydrogen dioxide solutions in various strengths from 6 per cent. upward.

Eye diseases, where there is a purulent external inflammation, are constantly being benefited by this agent. The Wills Eye Hospital, Philadelphia, uses a 50 per cent. strength of the so-called 15 volume solution. Blepharitis marginalis is quickly cured by touching the edges of the lids once or twice daily with a strong solution, care being taken to avoid getting it into the eye.

Ulcers of all kinds improve rapidly under its use, and for treating and cleansing venereal sores, as chancroids, etc., it is of great service.

Empyema, especially where there is from the first a stinking sanious exudation following incision, is very satisfactorily treated by washing out the cavity with a solution from one-half to full strength.

In appendicitis, the abscess cavity is cleansed with this solution by many operators, in preference to any other antiseptic. Robert T. Morris, of New York, has laid special stress on the value of the peroxide in these cases.

In follicular tonsilitis, the use of a spray, diluted just enough to prevent the smarting sensation, and alternating with this, one of the alkaline antiseptic sprays, or gargles, is a very satisfactory procedure.

Diphtheria and all naso-pharyngeal inflammations where there is a pseudo-membranous and septic condition, have been treated very widely by means of this agent. I like the plan of Jennings in Detroit, who uses an irrigation of an aqueous solution of one-eighth each of hydrogen dioxide and listerine. He throws the solution into the pharynx with an all-soft rubber syringe every one, two or three hours. The plan is an admirable one for treating children, and the combination is pleasant and effective.

Atrophic rhinitis is benefited remarkably by the use of a 40 per cent. spray. It should be used a few minutes before the employment of the usual alkaline, stimulating spray, and the powder insufflations. In this way the scabs are loosened, muco-purulent secretions are dissolved, and a stinking breath is converted into one that is pure and sweet.

In acute cases of eczema of the leg, we find this agent of the utmost value. The tissues are inflamed, hot, swollen and oozing, the itching is almost unendurable, the odor is offensive. To secure the best results the limb is elevated, and a diluted solution of the peroxide is applied frequently, with cheese cloth, gauze or an atomizer. In two or three days a marked change for the better will be apparent, the pruritus is allayed, the purulent exudation is checked, and all inflammatory symptoms are subsiding. At this stage we begin the use of a soothing ointment, such as the boracic acid or zinc oxide, using lime liniment to wash the parts instead of water. Under this treatment, combined with rest, we will see our patient rapidly cured.

Eczema of the anus will rapidly improve if the fissures are touched twice a day with this solution, then dried gently with cotton, and a glycerite of lead application made. In nearly every form of acute eczema in the first and second stages the peroxide will give us the keenest satisfaction. The regular solution is diluted with two or more parts of water. Hydrogen peroxide is an excellent anti-pruritic, and for this purpose it is widely used.

The hæmostatic value of this drug, as pointed out by Dr. Emerson Brewer, of New York, I can endorse. In operations on the nose and throat I have upon two occasions been enabled to check a persistent hemorrhage, when Monsel's solution and plugging had failed. At present I am in the habit of applying the full strength hydrogen peroxide after every operation on these parts. It is of special value after sawing out a deviated septum.

For flushing out a mammary abscess cavity this agent is invaluable.

Applied to the cervix uteri, adherent mucus is removed and our medications can be applied.

When it is inadvisable or impossible to make a complete opening of a fissure or abscess, irrigation with the peroxide will be found superior to all other antiseptics.

We have in peroxide of hydrogen a prompt, safe and efficient germicide. By its oxidizing power it rapidly decomposes pus, diphtheritic membranes, and other morbid putrefying material. It is a thorough deodorizer, and as a cleansing agent for foul wounds, abscesses, etc., it has no equal.

Of the different preparations of peroxide, Marchand's has been most uniformly satisfactory.

Since writing the foregoing paper my attention has been called to hydrozone, a stronger solution of peroxide of hydrogen, which, for some months, I have been using with much satisfaction.

